# ‘My late husband Mr Anua was a hard-working man’

**DOI:** 10.1098/rstb.2008.4001

**Published:** 2008-11-27

**Authors:** Andemba Anua

**Affiliations:** Ketabi Village, c/o Papua New Guinea Institute of Medical ResearchPO Box 60, Goroka, EHP 441, Papua New Guinea

I was approximately 12–13 years old when Dr Carleton Gajdusek came into the Purosa Valley to do research on kuru. My late husband Mr Anua was a hard-working man. He helped Carleton and Michael Alpers in their fieldwork. Sometimes when he was asked to go to other villages to report on kuru patients, I had to accompany him and we both went out. I have seen more of the villages to the south and west of Okapa than any of the other women in my village.

Many of Mr Anua's workmates retired from this arduous rural employment but he kept on working in the field—when death approached him and took his life. I am grateful to those who helped him to have a family and live the life in his village that he preferred.

I myself have also been helping Jerome Whitfield and his staff when they travel down to the Purosa Valley to collect the histories and cultural practices of the Pamusa people and study kuru patients.

I am proud to see that some of our children, including my own sons Sena and Tuli, have been employed by the Kuru Project like their fathers.

